# Feasibility and effectiveness of communication tools for addressing intimacy and sexuality in patients with cancer: a systematic review

**DOI:** 10.1007/s00520-024-08308-6

**Published:** 2024-01-17

**Authors:** Susanne A. M. Arends, Carlijn E. van Rossum, Corien M. Eeltink, Jantien E. Robertus, Linda J. Schoonmade, Anneke L. Francke, Irene P. Jongerden

**Affiliations:** 1 Department of Public and Occupational Health, Amsterdam UMC, Vrije Universiteit Amsterdam, Van Der Boechorststraat 7, NL-1081 BT Amsterdam, the Netherlands; 2 Amsterdam Public Health Research Institute, Van Der Boechorststraat 7, NL-1081 BT Amsterdam, the Netherlands; 3 Center of Expertise in Palliative Care, VU University Medical Center, Amsterdam, the Netherlands; 4 Oncology Daycare Center, Dijklander Hospital, Hoorn, the Netherlands; 5 Medical Library, VU University Amsterdam, Amsterdam, the Netherlands; 6 NIVEL. Netherlands Institute for Health Services Research, Utrecht, the Netherlands

**Keywords:** Communication, Oncology, Sexuality, Intimacy, Quality of life, Quality of care

## Abstract

**Purpose:**

Patients diagnosed with cancer might experience changes in intimacy and sexuality due to the illness itself, treatment, or psychological and social factors. Healthcare professionals (HCPs) often feel reluctant to discuss these changes. This study aimed to provide an overview of the feasibility and effectiveness of communication tools that support communication regarding changes in intimacy or sexuality among patients with cancer.

**Methods:**

This is a systematic review. Databases are PubMed, Embase, CINAHL, PsycInfo, Web of Science and Cochrane Library from inception to June 2023. The Mixed Methods Appraisal Tool was used to assess included studies. Data were summarized in data charting forms.

**Results:**

In total 35 studies were included, published between 2001 and 2023. Most had a quantitative design and moderate methodological quality. In 11 studies, the PLISSIT model (Permission, Limited Information, Specific Suggestions, Intensive Therapy) was used. Tools were integrated in counselling sessions or training programmes for individual patients, couples, groups of patients, or HCPs. All tools were considered feasible by patients or HCPs. Twenty studies reported significant improvement in sexual functioning, quality of life, quality of care or combined outcomes.

**Conclusion:**

Tools to support communication about changes in intimacy and sexuality among patients with cancer seem feasible and effective. The most commonly used tool, the PLISSIT model, proved to be feasible for HCPs and to have a positive effect on patients’ and partners’ sexual functioning and quality of life. Giving attention to changes in intimacy and sexuality seems to be important in itself, regardless of the communication tool or approach used.

**Supplementary Information:**

The online version contains supplementary material available at 10.1007/s00520-024-08308-6.

## Introduction

Cancer and its treatment may cause changes regarding intimacy and sexuality in patients [1–3]. Sexuality can be described as a central aspect of human beings that encompasses sexual self-concept, sexual functioning and sexual relationships [1, 4, 5]. In addition, sexuality can refer to sexual health, sexual pleasure, sexual awareness, self-esteem and sexual orientation [5–9]. Intimacy can be defined as an interactive process that occurs when a person discloses self-relevant feelings or information, resulting in feeling understood, cared for and accepted by the reaction of the other individual involved in the interaction [10, 11]. Moreover, intimacy is characterized as a quality of an interpersonal relationship in which individuals have reciprocal feelings of trust, connectedness, caring and emotional closeness and are able to openly communicate their thoughts and feelings with each other [12]. Intimate relationships can involve both emotional intimacy (e.g. sharing thoughts and feelings) and physical intimacy (e.g. touching and closeness) [12].

Changes in intimacy and sexuality can arise due to the cancer itself, the treatment or psychological and social factors such as anxiety or distress [13]. For instance, cancer treatment might cause problems with sexual desire and arousal problems [14–16]. Changes in sexuality can persist for years and can be extremely distressing for patients with cancer and their intimate partners [17].

Patients with cancer or patients who survived cancer have reported a need for information regarding changes in intimacy and sexuality [6]. Addressing sexual health adequately and timely, e.g. before, during and after treatment, may be crucial in identifying sexual problems and might ensure appropriate treatment and support [18, 19]. Discussing these issues can alleviate anxiety and psychological distress [20–22] and may improve the quality of sexual or intimate life [18, 22, 23]. In addition, failure to address the need for information can be associated with increased psychological morbidity, including depression and relational dissatisfaction, as well as a reduction in self-efficacy and overall quality of life [24].

Though these are important issues, healthcare professionals often feel reluctant to discuss changes in intimacy and sexuality, and they frequently remain unaddressed in cancer care [2, 17, 25–28]. Several barriers faced by healthcare professionals have been recognized, such as a lack of time, lack of privacy [29], feeling uncomfortable discussing the topic, the patient’s advanced age [30] and a lack of skills, training and knowledge [1, 22, 27, 31].

Research suggests that communication tools may help overcome barriers and help healthcare professionals to initiate a conversation with patients about changes in intimacy and sexual concerns [32–35]. These tools can consist of models, guides or training that support communication with patients about intimacy and sexuality. In a previously conducted narrative review, several specific tools were identified that address sexual health issues in terminally ill patients, including the stepwise PLISSIT model (Permission, Limited Information, Specific Suggestions, Intensive Therapy) [22]. However, this study did not address the effectiveness or feasibility of the identified tools [22]. Consequently, the question of which tools are effective and feasible for addressing changes in intimacy and sexuality remains unanswered.

In order to answer this question, we sought to provide an overview of the feasibility and effectiveness of tools that support communication regarding changes in intimacy or sexuality with patients with cancer in inpatient and outpatient care. We address the following review questions in this review:

1. What evidence exists for the feasibility of these tools, from the perspectives of the professionals and patients involved?
2. What evidence exists for the effectiveness of tools that support communication about concerns and needs regarding intimacy or sexuality with patients with cancer?
3. What differences are found between patients with advanced cancer, patients with early stage cancer and cancer survivors in the feasibility and effectiveness of these communication tools?

## Methods

### Design

We conducted a systematic review to identify studies that focused on tools that support communication between patients and healthcare professionals about concerns regarding intimacy or sexuality. The review was reported according to the Preferred Reporting Items for Systematic Reviews and Meta-Analyses (PRISMA) [36], and the protocol is registered in PROSPERO (CRD42021283852).

### Search strategy

A comprehensive search was performed in collaboration with a medical librarian (LS) in the bibliographic databases PubMed, Embase.com, Cinahl (Ebsco), APA PsycInfo (Ebsco), the Web of Science Core Collection and the Cochrane Library (Wiley) from inception to 26 June 2023. The search strategy consisted of free-text terms and controlled vocabulary for ‘cancer’ and ‘sexuality’ or ‘intimacy’ and ‘communication’. A search filter was used to exclude children. No language or time restrictions were included in the search strategy. In addition, the reference lists of all included studies were screened for relevant records. Duplicate articles were excluded (LS) using Endnote X20.01 (Clarivate™). The full search strategies are provided in Supplemental Material III.

### Eligibility criteria

We included all types of empirical quantitative, qualitative or mixed-methods studies that focus on the following:

- Tools (including models, guides, educational programmes or interventions) that promote and/or support communication between healthcare professionals and patients about concerns and needs regarding intimacy and sexuality.
- Adult patients (18 years of age or older) with any type and stage of cancer, including cancer survivors.
- Inpatient or outpatient settings.
- Outcomes regarding the effectiveness or feasibility of the aforementioned communication tools. More specifically, these were outcomes directly related to sexuality, quality of life (i.e. physical, psychological, social or spiritual aspects) or quality of care (i.e. perceived quality of care or perceived satisfaction with communication). Outcomes regarding feasibility included perceptions and experiences related to usability, willingness to participate, duration of the applied intervention and lost to follow-up.

In the first selection procedure we came upon studies that included mixed populations (patients with various diseases). In addition to the above criteria and after discussion in the research group, we decided to exclude studies that involved mixed populations if it was not possible to extract data for only patients with cancer.

### Study selection

**Fig. 1:**
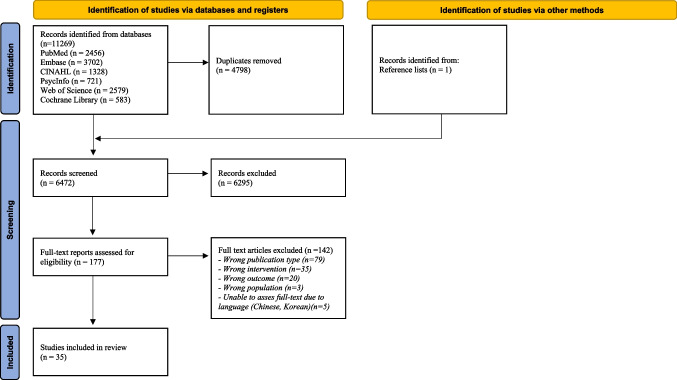
PRISMA flowchart [36]

Titles and abstracts were independently screened for potential eligibility by two reviewers (IJ and SA or CR or JR), using the systematic review software Rayyan (2016). Subsequently, two authors (IJ and SA or CR or JR) screened the full texts for eligibility. Any disagreement between authors was resolved by discussion with two other researchers (CE, AF). The study selection procedure and results can be found in the flow diagram in Fig. 1.

### Quality assessment

The methodological quality of the included studies was assessed independently by two reviewers (SA and CR or JR or IJ) using the Mixed Methods Appraisal Tool version 2018 (MMAT) [37]. The MMAT tool is designed to appraise the methodological quality of quantitative, qualitative and mixed-method studies [37]. Each study was evaluated using five quality items, depending on the study design. The items were rated by answering ‘Yes’ (green), ‘No’ (red) or ‘Can’t tell’ (orange). The latter category meant that the paper did not report enough information to answer ‘Yes’ or ‘No’. We did not contact authors for additional clarification; however, we did look for subsequent papers that might provide an answer. Any disagreement between reviewers was resolved by discussion and with the involvement of an additional reviewer (IJ). MMAT scores are presented as global scores (Supplemental I) and are indicated by stars (*) in the tables. The stars correspond to the number of questions answered with ‘Yes’ (Table 1).

**Table 1:** Study demographics

First author, year	Country	Study design	Study objective	Study population	MMAT^1^
Akeflo, 2022 [38]	Sweden	Quantitative non-randomized	Explore the effect of a nurse-led intervention on sexual health and well-being	260 female pelvic cancer survivors Mean age: 62.5 (SD^2^ 11.7)	*
De Almeida, 2020 [39]	Brazil	Quantitative non-randomized	Efficacy of PLISSIT^3^ on sexual function and QoL^4^	23 female breast cancer survivors Mean age: 54.6 (SD 7.1)	***
Bingham, 2022 [40]	UK	Quantitative non-randomized	Assess the acceptability and usability of an eLearning resource for HCPs^5^ and the potential impact on HCPs’ attitudes and beliefs	89 HCPs, of which 40 nurses, working in routine oncological care	**
Bokaie, 2022 [41]	Iran	Quantitative non-randomized	Effectiveness of group counselling on women’s sexual function and satisfaction after mastectomy surgery	32 married women after mastectomy Mean age: 39.8 (SD 7.5)	***
Bokaie, 2023 [42]	Iran	RCT^6^	Investigating the effect of online counselling in improving sexual quality of life	80 married women with breast cancer undergoing treatment Mean age: 40.6 (SD 4.1)	***
Chambers, 2015 [43]	Australia	RCT (3 arm)	Efficacy of peer-delivered telephone support versus nurse-delivered telephone counselling versus usual care	189 hetero-sexual men diagnosed with prostate cancer and their partners Mean age of men: 62.7 (SD 6.8) Mean age of partners: 59.8 (SD 7.4)	**
Chow, 2014 [44]	China	Mixed method	Feasibility of implementing a psycho-educational intervention programme for gynaecological cancer patients	26 women newly diagnosed with gynaecological cancer or scheduled for first-line surgery treatment Mean age: intervention 51.4 (SD 8.0); control 57.7 (SD 13.4)	**
Cullen, 2021 [45]	Canada	Qualitative	Developing and evaluating an online relational intimacy and sexual enhancement intervention for couples experiencing sexual difficulties	18 women diagnosed with breast cancer and their male partners Mean age: women 46.5 (SD 7.3) years; partners 47.4 (SD 8.2)	**
Du, 2020 [46]	China	RCT	The effect of empowerment education based nursing interventions on the post-operative sexual function and depression state of cervical cancer patients	69 married women diagnosed with cervical cancer Mean age: intervention 34.2 (SD 10.2); control 36.6 (SD 11.4)	**
DuHamel, 2016 [47]	USA	RCT	Efficacy of a telephone-based cancer survivorship intervention sexual health	82 female rectal cancer or anal cancer survivors Mean age: 55.4 (SD 11.6)	*
El-Sayed Saboula, 2015 [48]	Egypt	Quantitative non-randomized	Effectiveness of PLISSIT Counselling Model on female sexuality, body image and couple satisfaction for breast cancer women undergoing treatment	85 married women diagnosed with breast cancer Mean age: 43.1 (SD 10.0)	**
Esmkhani, 2021 [49]	Iran	RCT	Effect of individual therapy (PLISSIT) versus group therapy (Sexual Health Model) on Quality of Life in women with breast cancer	75 married women diagnosed with breast cancer Mean age PLISSIT group 38.1 (SD 5.5); SHM group 40.4 (SD 7.2); control 41.0 (SD 4.4)	**
Faghani, 2016 [50]	Iran	Quantitative non-randomized	Determine the effect of sexual rehabilitation using the PLISSIT model in post-mastectomy breast cancer survivors	100 married women, breast cancer survivors Mean age: 43.2 (SD 4.6)	***
Fatehi, 2019 [51]	Iran	RCT	Effects of intervention through psychosexual counselling on the sexual life quality	100 married women, breast cancer survivors Mean age intervention 44,8 (SD 6,7); control 43.8 (SD 6.6) years	****
Jonsdottir, 2021 [52]	Iceland	Quantitative non-randomized	Evaluate the benefits of a couple-based intervention focusing on sexual concerns	60 women in active cancer treatment and their partners; most patients (76.7%) diagnosed with breast cancer Mean age 52.0 (SD 10.7); partners 54.3 (SD 10.8)	***
Jonsdottir, 2021 [53]	Iceland	Quantitative non-randomized	Evaluate the effectiveness of a strengths-oriented therapeutic conversation intervention on confidence about how illness beliefs affect sexuality and intimacy on a perceived relationship quality	60 women in active cancer treatment and their partners; most patients (76.7%) diagnosed with breast cancer Mean age: women 52.0 (SD 10.7) years; partners 54.3 (SD 10.8)	***
Keshavarz, 2021 [54]	Iran	Quantitative non-randomized	Determining the effect of PLISSIT based counselling on sexual function, QoL and sexual distress	65 married women, breast cancer survivors Mean age: 43.4 (SD 5.6)	***
Khoei, 2022 [55]	Iran	RCT (3 arm)	Effect of PLISSIT versus Grouped Sexuality Education (GSE) on self-reported sexual behaviours experienced by women after breast cancer diagnoses	75 married women diagnosed with breast cancer Mean age PLISSIT group 38.1 (SD 5.6); GSE group 40.4 (SD 7.3); control 41.0 (SD 4.4)	**
Li, 2023 [56]	Hong Kong	RCT	Investigate the effectiveness of a WeChat couple-based psychosocial support platform in promoting sexual functioning, relationship satisfaction and quality of life	98 women newly diagnosed with gynaecologic cancer and their partners Mean age women 46.0 (SD 7.9) years; partners 48.1 (SD 8.1)	**
Maughan, 2001 [57]	UK	Mixed methods	To evaluate the effect of an innovative psychosexual intervention by a clinical nurse specialist in relation to the specific areas of quality of life and sexual function	36 women diagnosed with gynaecological cancer Mean age 50 (range 15–78)	*
McCaughan, 2020 [19]	UK	Mixed methods	To assess feasibility, acceptability and satisfaction with the tablet-based Engagement, Assessment, Support and Sign-posting (EASSi) tool	Eight HCPs, of which three nurse specialists and one well-being nurse, using the tool during 89 patient appointments	**
McCaughan, 2021 [58]	UK	Mixed methods	Evaluates a brief e-learning resource designed to improve sexual well-being support and examine its effects on healthcare professionals’ sexual attitudes and beliefs	44 HCPs, of which 31 nurses, working in the area of prostate cancer care	*
Mohammadi, 2022 [59]	Iran	RCT	Investigate the effect of counselling based on the EX-PLISSIT (Extended-PLISSIT) model on improving sexual function and sexual quality of life	110 women who survived gynaecologic cancer Mean age intervention 40.4 (SD 9.0); control 40.5 (SD 10.8)	****
Olcer, 2022 [60]	Turkey	RCT	To determine the effect of BETTER^7^ model-based counselling programme on sexual problems of women with breast cancer	60 women diagnosed with breast cancer Mean age intervention 40.6 (SD 4.7); control 40.0 (SD 4.4)	*****
Perz, 2015 [61]	Australia	RCT	To evaluate the early stages of the PLISSIT model by examining the relative efficacy of written information provision about cancer related sexual changes, and information provision accompanied by a single session of counselling, for people with cancer and their partners	88 patients diagnosed with cancer (41 male, 47 female) Mean age 52.7 (SD 11.5)	*
Reese, 2012 [62]	USA	Quantitative non-randomized	Assess the feasibility and efficacy of a Telephone-Based Couples Intervention for physical intimacy and sexual concerns in colorectal cancer	Nine patients diagnosed with colorectal cancer (five male, four female) and their partners Mean age 61.6 (SD 14.5)	**
Reese, 2019 [34]	USA	Mixed methods	Examining the feasibility, acceptability, and potential benefits of a novel intervention, improving Sexual Health and Augmenting Relationships through Education (iSHARE) for breast cancer clinicians	Seven outpatient clinicians working in breast cancer care with audio recordings of clinical encounters with 134 breast cancer patients Mean age of patients 58.3 (SD 11.1)	*
Reese, 2021 [63]	USA	RCT	Evaluate a multi-media intervention called Starting the Conversation (STC) aimed at facilitating breast cancer patients ‘ clinical communication about sexual health	144 women diagnosed with breast cancer Mean age: STC group 55.8 (SD 11.2); control 56.1 (SD 10.9)	***
Reese, 2023 [64]	USA	RCT	To assess feasibility and acceptability of an intervention to promote communication about sexual health and to assess the effect of this intervention on patients’ sexual and psychological health outcomes	32 women diagnosed with any stage of gynaecologic cancer Mean age 62.2 (SD 9.9)	***
Roberts, 2020 [65]	Australia	Quantitative non-randomized	Assess the impact of a screening tool, the Brief Sexual Symptom checklist for Women, on referral rates to physiotherapist, sexual counsellors, and psychologist for sexual issues	318 women survivors of gynaecological cancer Mean age: intervention 56.2 (SD 12.6); control 63.2 (SD 13.8)	**
Shalamzari, 2022 [66]	Iran	RCT	Comparing the effectiveness of sexual counselling based on the BETTER and PLISSIT models on the quality of sexual life	80 women with breast cancer after mastectomy Mean age: intervention (BETTER) 41.3 (SD 4.6); Control (PLISSIT) 42.2 (SD 4.3)	****
Taleb, 2023 [67]	Yemen	Quantitative non-randomized	To determine the effect of nursing interventions on urinary, bowel and sexual dysfunction	30 men who underwent radical prostatectomy Eight participants (26.7%) aged < 55 years; 22 participants (73.3%) aged > 55 years	***
Wang, 2022 [68]	Canada	Mixed method	Support medical radiation therapists (MRT) in starting sexual health conversations with cancer patients with the use of sexual health champions and targeted training	19 radiation therapist “champions” 135 radiation therapists 599 patients with cancer in radiation therapy No information about patient or HCP age	-
Winterling, 2020 [23]	Sweden	Qualitative	Evaluate the Fex-talk intervention	140 nurses who work with cancer patients No information about age	**
Zhang, 2022 [69]	China	RCT	To explore the effectiveness of a nurse-led couples intervention on the marital quality of patients with gynaecological cancer and their husbands	95 women diagnosed with gynaecological cancer and their husbands Mean age of women: intervention group 49.9 (SD 5.5); control 48.6 (SD 7.5)	*****

Abbreviations:

^1^*MMAT* Mixed Methods Appraisal Tool

^2^*SD* Standard Deviation

^3^*PLISSIT* Permission, Limited Information, Specific Suggestions, Intensive Therapy

^4^*QoL* Quality of Life

^5^*HCPs* Health Care Professionals

^6^*RCT* Randomized Controlled Trial

^7^*Better* Bring up the topic, Explain, Telling, Timing, Education, Recording

### Data extraction

Three researchers (SA, CR, IJ) independently extracted data from the included studies in a predefined form. The following study characteristics were extracted: year of publication, country, study design, study population (including number of participants and age) and communication tool. For feasibility, where applicable, we extracted data on usage, willingness to participate, lost to follow-up and duration of the intervention. For effectiveness, we extracted data on outcomes related to intimacy and sexuality, quality of life and quality of care that were measured in patients, their partners or healthcare professionals.

### Data synthesis

We decided beforehand not to do a meta-analysis because of expected heterogeneity in design and outcomes. Instead, we synthesized outcomes using a narrative approach and predesigned data charting forms. Data were extracted independently by two researchers (SA and CR or SA and IJ). The data charting forms were discussed with two other researchers (CE and AF). After several discussion rounds, all researchers agreed upon the finalized version of the charting tables.

## Results

### Results of the selection process

The literature search generated a total of 11,269 references. After removing duplicates, 6472 records were screened for eligibility based on title and abstract (Fig. 1). We excluded 6295 records, mainly because these studies did not focus on adult patients with cancer or did not include a tool supporting communication between healthcare professionals and patients. Five studies were excluded because they were written in Chinese or Korean and we were unable to read and assess the full text [70–74]. After reading the full text of 177 remaining references, we included 35 articles, published between 2001 and 2023. Of these, four articles concerned the same study, but were reporting different outcomes. Therefore, they were included as separate studies.

In total, 2567 patients (number per study ranging from 7 to 318), 677 partners (ranging from 7 to 189) and 423 healthcare professionals (ranging from 7 to 140), the majority being nurses (*n* = 215), were included in the studies. Most of the included patients in these studies were female (*n* = 2234, 87.0%). The mean age of the participating patients ranged from 34.2 to 62.7 years and of participating partners from 47.4 to 61.6 years (see Table 1). The country with the most studies was Iran (*n* = 9), followed by the USA (*n* = 5).

### Methodological quality of the studies

The designs used and evaluated, as identified by the MMAT tool [37], were randomized controlled trials (RCTs) (*n* = 15), quantitative non-randomized studies (*n* = 12), qualitative studies (*n* = 2) and mixed methods studies (*n* = 6). Quality assessment scores using the MMAT tool ranged from 0 to 5 out of 5 (Supplemental I). Only two RCTs had maximum scores (5 out of 5 items present). Six of the 35 studies had low scores (0 or 1 out of 5 items); two were RCTs and four were mixed methods studies.

### Tools supporting communication about intimacy and sexuality

Various tools were described in the 35 included studies, (see Table 1). The most commonly used tool was the specific, stepwise model PLISSIT [39, 48–50, 54, 61, 66, 75]. Two studies used the Extended PLISSIT model (EX-PLISSIT), which differs from the original PLISSIT model in that it is feedback-oriented while PLISSIT is a linear model [59, 68]. Other tools used were BETTER (Bring up the topic, Explain, Telling, Timing, Education, Recording), EASSi (Engagement, Assessment, Support and Signposting) or self-developed tools. Tools were applied in counselling sessions, educational programmes, intervention programmes and nurse-led interventions (see Supplemental II).

Thirteen of the 35 studies focused on tools that support communication with individual patients with cancer or cancer survivors. Nine studies used communication tools for couples (patients with cancer and their intimate partner). Five studies focused on the use of communication tools for groups of patients and two studies used communication tools for both individual patients and couples or groups. Communication tools for healthcare professionals, predominantly nurses and nurse specialists, were used in six studies. These tools were applied in training sessions for healthcare professionals to equip them for conversations with their patients (Supplemental II).

### Feasibility of communication tools

In 33 studies, feasibility was assessed with regard to duration, usability, willingness to participate and lost to follow-up (see Table 2).

**Table 2:** Outcomes regarding feasibility of tools supporting communication about intimacy and sexuality

First author, year	Tool (model, programme, intervention, components, duration)	Willingness to participate, including eligible and participating participants	Lost to follow-up, including compliance with programme	Usability	MMAT^1^
Akeflo, 2022 [38]	PLISSIT^2^ Nurse-led intervention with visits and/or phone calls or digital meetings, applied 3 months to several years (individualized care)	975 eligible participants of which 605 took part in baseline questionnaire (62.1%) 605 participants were included of which 379 (62.6%) approved to take part in the intervention	605 participant took part in baseline questionnaire of which 226 (37.4%) were lost to follow-up before the intervention 379 participants took part in the intervention of which 119 (31.4%) were lost to follow-up In total 345 out of 605 participants (57.0%) were lost to follow up	88.6% of the participants reported that they were moderately to very satisfied with the help offered regarding sexual health issues	*
De Almeida, 2020 [39]	PLISSIT counselling, five weekly sessions of 1.5 h each Control group received one lecture (2 h)	40 eligible patients of which 23 agreed to participate (57.5%)	23 patients included in study of which 5 were lost to follow-up, all from intervention group (21.7%)		***
Bingham, 2022 [40]	Engagement, Assessment, Support and Signposting (EASSi) framework, one-time training (eLearning), duration approximately 1 h		157 participants included in study of which 68 were lost to follow-up (43.3%, calculated upon post-test survey participation) 89 participants completed the study of which 2 did not fully comply with the intervention (2.4%)	Intervention considered easy to use by HCPs^3^	**
Bokaie, 2022 [41]	Problem-solving approach (sessions), counselling, eight weekly sessions, 90 min per session	100% participation rate None of the patients wanted to be in control group			***
Bokaie, 2023 [42]	Solution-focused approach, online counselling, eight weekly sessions, 90 min per session Control group: educational pamphlet to read every 3 weeks	165 eligible participants of which 80 agreed to participate (48.5%)	80 participants included in study of which 15 were lost to follow-up (18.8%)		***
Chambers, 2014 [43]	DVD and tip sheet and sessions, counselling, six–eight telephone sessions delivered by nurses (intervention 1) or by peers (intervention 2)	405 eligible participants of which 189 agreed to participate (46.7%)	189 participants included in study of which 30 were lost to follow-up or did not comply (15.9%)	Duration of sessions longer in the nurse group (36 min) as compared to the peer group (29 min) (*p*^4^ < .001)	**
Chow, 2014 [44]	Structured sessions, psycho-educational intervention programme, three individual sessions and one group session Control group received attention on 4 occasions	30 eligible participants of which 26 agreed to participate (86.7%)	26 participants included in study of which 2 were lost to follow-up (3.9%) Intervention: 13 participants included of which 4 did not fully comply (30.7%); Control: 13 participants included of which 7 did not receive all attention (53.8%)	Intervention considered appropriate (time, frequency and provider) by patients Considered practical and feasible by nurses	**
Cullen, 2021 [45]	Online couple-based psychosexual intervention, six sessions	25 eligible couples of which 18 agreed to participate (72%)	18 couples included in study of which 3 did not comply with the intervention programme (16.7%)		**
Du, 2020 [46]	Group intervention with education sessions, seven sessions of at least 30 min				**
DuHamel, 2016 [47]	Sessions and booster call, educational intervention, four sessions of 1 h, booster call between sessions Control group: no calls	82/204 (40%) eligible patients participated 204 eligible participants of which 82 agreed to participate (40%)	In total 82 participants included in study of which 9 were lost to follow-up; Intervention: 40 participants included of which 6 were lost to follow-up (15.0%); control: 42 participants of which 3 were lost to follow up (7.1%) Compliance with programme, 96% HCPs (manual), 89% patients (homework)		*
El- Sayed Saboula, 2015 [48]	PLISSIT counselling, six sessions lasting 2 h each during 3 weeks		85 participants included in study of which 19 were lost to follow-up (22.3%)		**
Esmkhani, 2021 [49]	PLISSIT (individual) and Sexual Health Model [SHM] (group), counselling, one–three sessions of 40–60 min (individual), 6-h workshop (group) Control group received usual care	395 eligible participants of which 75 agreed to participate (19%)	In total 75 participants included in study of which 10 were lost to follow-up (13.3%); Individuals: 25 participants of which 3 were lost to follow-up (12.0%); Group: 25 participants of which 0 were lost to follow-up; Control: 25 participants of which 7 were lost to follow-up (28.0%)		**
Faghani, 2016 [50]	PLISSIT counselling, 4 × 90-min sessions Control group: no intervention				***
Fatehi et al., 2019 [51]	Schover’s sexual assessment method, psychosexual counselling, six weekly sessions of 90–120 min	289 eligible participants of which 118 agreed to participate (40.8%)	In total 118 participants included in study of which 18 were lost to follow-up or excluded from study (15.2%); Intervention: 59 participants of which 8 lost to follow-up (13.6%); Control: 59 participants of which 10 lost to follow-up (16.9%)		****
Jonsdottir, 2021 [52]	Couple based Strengths-Oriented Therapeutic Conversation (CO-SOTC), three sessions of 45 min	149 eligible couples of which 60 agreed to participate (49% acceptance rate)	60 couples included in study of which 1 withdrew after randomization (1.7%)		***
Jonsdottir, 2021 [53]	Couple based Strengths-Oriented Therapeutic Conversation (CO-SOTC), three sessions of 45 min	149 eligible couples of which 60 agreed to participate (49% acceptance rate)	60 couples included in study of which 1 withdrew after randomization (1.7%)		***
Keshavarz, 2021 [54]	PLISSIT counselling, seven sessions of 60 min for 4 weeks	88 eligible participants of which 67 agreed to participate (76.1%)	2/67 (3.0%) were lost to follow-up 67 participants included in study of which 2 were lost to follow-up (3.0%)		***
Khoei, 2022 [55]	PLISSIT (individual) and Grouped Sexuality Education (group) counselling, one–three sessions of 40–60 min (individual) or 6-h workshop (group)	395 eligible participants of which 75 agreed to participate (19.0%)	In total 75 participants included in study of which 10 were lost to follow-up (13.3%); Individuals: 25 participants of which 3 were lost to follow-up (12.0%); Group: 25 participants of which 0 were lost to follow-up; Control: 25 participants of which 7 were lost to follow-up (28.0%)		
Li, 2023 [56]	Systematic Transactional Model of Stress and Coping; psychosocial intervention programme (WeChat), 8-week programme Control group: six articles received over an 8-week period	185 eligible patient-partner dyads of which 98 couples agreed to participate (53%)	98 patients included in study of which 26 were lost to follow-up (26.5%); Intervention: 49 patients of which 14 were lost to follow-up (28.6%); Control: 49 patients of which 12 were lost to follow-up (24.5%) 98 partner included in study of which 29 were lost to follow-up (29.6%); Intervention: 49 partners of which 14 were lost to follow-up (28.6%); Control: 49 partners of which 15 were lost to follow-up (30.6%)		**
Maughan, 2001 [57]	Information, advice and support, psychosexual intervention by Clinical Nurse Specialist, with visit prior to surgery and home visits (3 on average) Control group had no visits	Acceptance rate 100%			*
McCaughan, 2020 [19]	EASSi^5^ framework (Tablet-based) Training (e-learning) of 30 min to use tool			HCPs viewed the EASSi tool as feasible, acceptable, appropriate HCPs deemed EASSi unsuitable for patient who is medically unstable, for patient who is attending with a family member (other than partner), or who is ‘not concerned’ about sexual issues	**
McCaughan, 2021 [58]	EASSi framework, brief e-learning consisting of three sections			HCPs viewed tool as easy to use	*
Mohammadi, 2022 [59]	EX-PLISSIT^6^, Counselling, 4 weekly sessions lasting 60–90 min Control group: one online session	122 eligible participants of which 110 agreed to participate (90.2%)	110 participants included in study of which 11 were lost to follow-up (10.0%)		****
Olcer, 2022 [60]	BETTER^7^ Counselling, four sessions with 1-week interval	116 eligible participants of which 77 agreed to participate (66.4%)	77 participants included in study of which 12 were lost to follow-up (15.6%); Intervention: 38 participants of which 5 were lost to follow-up (13.2%); Control: 39 participants of which 7 were lost to follow-up (17.9%)		*****
Perz, 2015 [61]	PLISSIT Self-help booklet consisting of 68 pages providing self-help information (group) or information and 1-h telephone consultation (group 2)	394 eligible patients of which 88 agreed to participate (22.3%); 122 eligible partners of which 53 agreed to participate (43.4%)	In total 88 patients included in study of which 29 were lost to follow-up (32.9%); Group 1: 45 patients included of which 12 were lost to follow-up (26.7%); Group 2: 43 patients included of which 17 were lost to follow-up (39.5%) In total 53 partners included in study of which 22 were lost to follow-up (41.5%); Group 1: 25 partners of which 7 were lost to follow-up (28.0%); Group 2: 28 partners of which 15 were lost to follow-up (53.6%)	Self-help booklet and Health professional information were rated as useful in helping to manage changes to sexuality and talking with their partner about sexuality Self-help booklet was rated favourably	*
Reese, 2012 [62]	Intimacy Enhancement Intervention, Counselling, four phone-based sessions of 50 min	34 couples invited to participate of which 14 agreed to participate (41.2%)	14 couples included in study of which 5 were lost to follow-up of did not complete intervention (35.7%)	83% of patients perceived the programme as easy to participate in and helpful in communication and behavioural skills	**
Reese, 2019 [34]	PLISSIT Training to equip clinicians for counselling, two modules (15 and 60 min)	Eight eligible clinicians of which 7 participated (87.5%); 172 approached patients of which 137 agreed to participate (79.9%)	All clinicians completed the intervention; 137 patients included in study of which 3 were lost to follow-up (2.2%)	Clinicians’ responses on post intervention programme evaluation: The results suggested intervention feasibility	*
Reese, 2021 [63]	Starting the Conversation, Online patient training including video slideshow (20 min) and five-page workbook	177 eligible participants of which 144 agreed to participate (81.4%)	In total 144 participants included in study of which 22 were lost to follow-up or did not fully comply to intervention (15.2%)		***
Reese, 2023 [64]	Starting the Conversation, educational intervention for patients including video slideshow (23 min) and five-page workbook	42 eligible participants of which 32 agreed to participate (76.2%)	32 participants included in study of which 1 was lost to follow-up (3.1%)		***
Roberts, 2020 [65]	Brief sexual symptom checklist for women applied in routine care				**
Shalamzari, 2022 [66]	PLISSIT and BETTER-model, Counselling, four sessions of 60–90 min with 1-week interval		78 participants included in study of which 2 were lost to follow-up (2.6%)		****
Taleb, 2023 [67]	Structured sessions, nursing intervention consisting of five sessions				***
Wang, 2022 [68]	EX-PLISSIT applied in training for HCPs^8^				-
Winterling, 2020 [23]	Fex-talk, Educational intervention, single session lasting 2 h, optional second session			Fex-Talk intervention was experienced positively by the participating nurses, seemed to increase awareness and appeared to boost confidence to initiate discussions	**
Zhang, 2022[69]	Structured sessions, Nurse led intervention, monthly sessions of 4 h, lasting for three consecutive treatment cycles	120 eligible couples of which 106 agreed to participate (88.3%)	In total 106 couples included in study of which 11 were lost to follow-up (10.4%); Intervention: 53 couples of which 7 were lost to follow-up (13.2%); Control: 53 couples of which 4 were lost to follow-up (13.2%)		*****

Abbreviations:

^1^*MMAT* Mixed Methods Appraisal Tool

^2^*PLISSIT* Permission, Limited Information, Specific Suggestions, Intensive Therapy

^3^*HCPs* Healthcare Professionals

^4^*P P*-value

^5^*EASSI* Engagement, Assessment, Support and Signposting

^6^*EX-PLISSIT* Extended-PLISSIT

^7^*BETTER* Bring up the topic, Explain, Telling, Timing, Education, Recording

^8^*HCPs* Healthcare professionals

#### Duration of the intervention

The duration of the intervention programme for the tools, such as counselling sessions, home visits or educational programmes, ranged from one single session to eight sessions, with each session ranging from 30 up to 120 min (see Table 2).

#### Usability

Ten studies reported outcomes relating to usability. Overall, patients, partners and healthcare professionals experienced tools as useful in increasing awareness about the topic, valuable and helpful in addressing intimacy and sexuality, and simple to use. In one study, healthcare professionals considered the tablet-based Engagement, Assessment, Support and Signposting tool (EASSi) as less suitable when patients were medically unstable, were attending the appointments with a family member (other than their partner), or when patients were not concerned about sexual issues [19].

#### Willingness to participate

Data on willingness to participate could be extracted from 24 studies. Rates varied from 19% in two studies [49, 55] to 100% in two studies [41, 57]. Of the two studies where willingness to participate was 100%, one study involved a clinical nurse specialist intervention in individual patients [57] and the other study involved a problem-solving approach for groups of patients [41]. Both high and low rates of willingness to participate were found in individual (19% to 100%) and group interventions (19% to 100%). Two studies among healthcare professionals reported on willingness to participate (56.7% and 87.5%) [34, 40].

#### Lost to follow-up

In 25 studies, lost to follow-up or compliance rate was reported, indicating the willingness to continue participation in the programme or comply with the intervention. Lost to follow-up rates ranged from 1.7% in a study among couples to 57% in a study applying PLISSIT in individual patients. Lost to follow-up varied in studies with individual patients (2.0% to 57.0%), in couples (1.7% to 35.7%) and in groups of patients (10.0% to 21.7%).

### Effectiveness of communication tools related to intimacy and sexuality

Of the 35 publications, 34 reported outcomes that indicate effectiveness i.e. outcomes related to intimacy and sexuality, quality of life or quality of care.

#### Outcomes related to intimacy and sexuality

Twenty-two studies reported on outcomes related to intimacy and sexuality, i.e. sexual function, sexual distress, sexual concerns, sexual communication, sexual behaviour and sexual satisfaction (see Table 3). Besides sexual functioning, some studies also measured outcomes related to intimacy and sexuality, such as sexual self-confidence, sexual concerns, sexual beliefs and relationship satisfaction.

**Table 3:** Outcomes regarding effectiveness of tools supporting communication about intimacy and sexuality

Outcomes related to intimacy and sexuality				
First author, year	Tool (model, programme, intervention, components, duration)	Sexual functioning	Other outcomes related to intimacy or sexuality	MMAT^1^
Akeflo, 2022 [38]	PLISSIT^2^ Nurse-led intervention with visits and/or phone calls or digital meetings, applied 3 months to several years (individualized care)	Sexual functioning assessed with a self-developed questionnaire, reported reduced genital pain (*p*^3^ < .05) and decrease in ability to have an orgasm (*p* < .05) post intervention Majority of participants reported no changes in sexual functioning	Overall sexuality and sexual life assessed with a self-developed questionnaire, reported increased satisfaction with overall sexuality and sexual life post intervention (*p* < .05)	*
De Almeida, 2020 [39]	PLISSIT counselling, five weekly sessions of 1.5 h each Control group received one lecture (2 h)	Sexual function, assessed with FSFI^4^ (ns^5^)		***
Bokaie, 2022 [41]	Problem-solving approach (sessions), counselling, eight weekly sessions, 90 min per session	Sexual function assessed with FSFI, improved function at follow-up as compared to baseline (*p* < .001)	Sexual satisfaction assessed with Larson questionnaire, higher satisfaction at follow-up as compared to baseline (*p* < .01)	***
Chambers, 2015 [43]	DVD and tip sheet and sessions, counselling, six–eight telephone sessions delivered by nurses (intervention 1) or by peers (intervention 2)	Sexual function assessed with IIEF^6^ (men, ns) or FSFI (women, ns)	Utilization of erectile dysfunction treatments, in intervention groups higher as compared to control (*p* < .05) Sexual self-confidence assessed with the Psychological Impact of Erectile Dysfunction – Sexual Experience (ns) Marital satisfaction assessed with the Revised Dyadic Adjustment Scale (ns)	**
Cullen, 2021 [45]	Online couple based psychosexual intervention, six sessions		The intervention programme deepened emotional intimacy and connection. Also, it improved sexual relationship and sexual communication (qualitative data)	**
Du, 2020 [46]	Group intervention with education sessions, seven sessions of at least 30 min	Sexual function assessed with FSFI, improved function in the intervention group as compared to control (*p* < .05)		**
DuHamel, 2016 [47]	Sessions and booster call, educational intervention, four sessions of 1 h, booster call between sessions Control group: no calls	Sexual function assessed with FSFI (ns)		*
El- Sayed Saboula, 2015 [48]	PLISSIT, counselling, six sessions lasting 2 h each during 3 weeks	Sexual function assessed with FSFI, improved function post intervention as compared to baseline (*p* < .05)	Couple satisfaction assessed with Revised dyadic adjustment scale, improved satisfaction post intervention (*p* < .001)	**
Faghani, 2016 [50]	PLISSIT Counselling, 4 × 90-min sessions Control group: no intervention	Sexual function assessed with FSFI, improved function in the intervention group as compared to control (*p* < .05) and improved function post intervention as compared to baseline in the counselling group (p < .01)		***
Fatehi, 2019 [51]	Schover’s sexual assessment method, psychosexual counselling, six weekly sessions of 90–120 min	Sexual function assessed with FSFI, improved function in intervention group as compared to control (*p* < .001)	Sexual satisfaction assessed with Larson Inventory of Sexual Satisfaction (ISS) (ns)	****
Jonsdottir, 2021 [52]	Couple based Strengths-Oriented Therapeutic Conversation (CO-SOTC), three sessions of 45 min		Sexual concerns assessed with Sexual Concern Questionnaire (SCQ), reduced concerns post intervention as compared to baseline (*p* < .05)	***
Jonsdottir, 2021 [53]	Couple based Strengths-Oriented Therapeutic Conversation (CO-SOTC), three sessions of 45 min		Quality of relationship assessed with Partnership Questionnaire, improved quality post intervention as compared to baseline in women and partners (*p* < .05)	***
Keshavarz et al., 2021 [54]	PLISSIT Counselling, seven sessions of 60 min for 4 weeks	Sexual function assessed with FSFI, improved function post intervention as compared to baseline (*p* < .01)	Sexual distress assessed with FSDS-R^7^, reduced distress post intervention as compared to baseline (*p* < .01)	***
Khoei, 2022 [55]	PLISSIT (individual) and Grouped Sexuality Education (group) Counselling, one–three sessions of 40–60 min (individual) or 6-h workshop (group)		Sexual behaviour assessed with Sexual Behaviour Questionnaire: - Between groups: improved sexual behaviour in GSE^8^-group as compared to control (*p* < .001) - Within groups: Improved sexual behaviour in GSE group and in control group post intervention as compared to baseline (*p* < .001) - PLISSIT group (ns)	**
Li et al., 2023 [56]	Systematic Transactional Model of Stress and Coping; psychosocial intervention programme (WeChat), 8-week programme Control group: six articles received over an 8-week period	Sexual function assessed with FSFI (ns)	Relationship satisfaction assessed with Chinese version of the Revised Dyadic Adjustment Scale (CR-DAS), indicating significant improvement post intervention as compared to baseline for both patients (*p* = .001) and partners (p < .05)	**
Maughan, 2001 [57]	Information, advice and support, psychosexual intervention by Clinical Nurse Specialist, with visit prior to surgery and home visits (3 on average) Control group had no visits	Sexual functioning assessed with Lasry Sexual Functioning Scale (ns)		*
Mohammadi, 2022 [59]	EX-PLISSIT^9^, Counselling, four weekly sessions lasting 60–90 min Control group: one online session	Sexual function assessed with FSFI, significantly higher overall sexual function post intervention compared to baseline in both groups (*p* < .001)		****
Olcer, 2022 [60]	BETTER^10^ Counselling, four sessions with 1-week interval	Sexual functioning assessed with FSFI, improved functioning after counselling as compared to control group after 4 weeks (*p* < .05)	Relationship satisfaction measured with the Brief Dyadic Adjustment Scale (BDAS), in self-help group improved dyadic adjustment and a lower level of distress as compared to baseline (*p* < .05) Satisfaction with sexual relationship assessed with one question (ns)	*****
Perz, 2015 [61]	PLISSIT Self-help booklet consisting of 68 pages providing self-help information (group) or information and 1-h telephone consultation (group 2)	Sexual function assessed with the Changes in Sexual Functioning Questionnaire (CSFQ-14)(ns)		*
Reese, 2012 [62]	Intimacy Enhancement Intervention, Counselling, four phone-based sessions of 50 min	Sexual function assessed with FSFI (female) and IIEF (men), indicating improved female functioning post intervention as compared to baseline (effect size 1.15) and little improvement in male functioning	Sexual distress assessed with the Index of Sexual Satisfaction, indicating decrease in sexual distress post intervention as compared to baseline (effect size − 1.01) Intimacy assessed with Miller Social Intimacy Scale, indicating little improvement Dyadic adjustment assessed with Dyadic Adjustment Scale, indicating medium effect size	**
Reese, 2021 [63]	Starting the Conversation, Online patient training including video slideshow (20 min) and five-page workbook	Sexual function assessed with items from the PROMIS SexFS^11^ Brief Profile version 2.0, indicating more women being sexually active in intervention group as compared to control (*p* < .05)		***
Taleb, 2023 [67]	Structured sessions, nursing intervention consisting of 5 sessions	Erectile function assessed with the Sexual Health Inventory for Men (SHIM), indicating improved sexual function post intervention (p < .05)		***
Outcomes related to quality of life				
First author, year	Tool (model, programme, intervention, components, duration)	Quality of Life overall	Related to quality of life	MMAT
Akeflo, 2022 [38]	PLISSIT Nurse-led intervention with visits and/or phone calls or digital meetings, applied 3 months to several years (individualized care)	Quality of life assessed with several items in a self-developed questionnaire, reported significantly increased level of quality of life post intervention (*p* < .001), significant reduced depressive mood (*p* = .003) and significant reduction of anxious mood (*p* < .001)		*
De Almeida, 2020 [39]	PLISSIT counselling, five weekly sessions of 1.5 h each Control group received one lecture (2 h)	Quality of life, assessed with WOHQOL-BREF^12^ (ns)		***
Bokaie, 2023 [42]	Solution-focused approach, online counselling, eight weekly sessions, 90 min per session Control group: educational pamphlet to read every 3 weeks	Sexual quality of life assessed with the Sexual Quality of Life (SQL) Questionnaire, significant increase in sexual quality of life post intervention as compared to baseline (*p* < .05)		***
Chow, 2014[44]	Structured sessions, psycho-educational intervention programme, three individual sessions and one group session Control group received attention on four occasions	Quality of life assessed with TCHI FACT-G^13^ (ns)		**
Du, 2020 [46]	Group intervention with education sessions, seven sessions of at least 30 min	Quality of life assessed with EORTCQ-QLQ-C30^14^ (ns)	Depression assessed with the self-rating depression scale (SDS), improved depression scores in intervention and control group, with lower scores in Intervention group as compared to Control group (*p* < .05)	**
DuHamel, 2016 [47]	Sessions and booster call, educational intervention, four sessions of 1 h, booster call between sessions Control group: no calls	Quality of life assessed with EORTC-QLQ-C30 (ns)		*
El- Sayed Saboula, 2015 [48]	PLISSIT, counselling, six sessions lasting 2 h each during 3 weeks		Side effects of treatment, reduced side effects regarding nausea and vomiting, diarrhoea and pain post intervention (*p* < .05) Body image assessed with Body Image Scale, improved post intervention (*p* < .05)	**
Esmkhani, 2021 [49]	PLISSIT (individual) and Sexual Health Model [SHM] (group), counselling, one–three sessions of 40–60 min (individual), 6-h workshop (group) Control group received usual care	Quality of life assessed with EORTC QLQ C30 (ns)		**
Faghani, 2016 [50]	PLISSIT Counselling, 4 × 90-min sessions Control group: no intervention	Quality of life assessed with the Sexual Quality of Life-Female (SCOL-F)(ns)		***
Fatehi, 2019 [51]	Schover’s sexual assessment method, psychosexual counselling, 6 weekly sessions of 90–120 min	Quality of life assessed with Sexual Quality of Life-Female (SCOL-F), with higher levels of sexual quality of life in intervention as compared to control at follow-up (91.0 and 38.2, respectively; *p* < .001)		****
Keshavarz, 2021 [54]	PLISSIT counselling, 7 × 60-min sessions	Quality of life assessed with WHOQOL-BREF: Improved quality of life post intervention (*p* < .01)		***
Li, 2023 [56]	Systematic Transactional Model of Stress and Coping; psychosocial intervention programme (WeChat), 8-week program Control group: six articles received over an 8-week period	Quality of life assessed with the Functional Assessment of Cancer Therapy-General (FACT-G), significant improvement for patients in overall quality of life as compared to baseline (*p* < .05). Also significant improvement in subdomains physical well-being (*p* < .05), social well-being (*p* < .05) and functional well-being (*p* < .05) Partners in the intervention group only had significant improvement on the subdomain physical health (*p* < .05) compared to control group		**
Maughan, 2001 [57]	Information, advice and support, psychosexual intervention by Clinical Nurse Specialist, with visit prior to surgery and home visits (3 on average) Control group had no visits	Quality of life assessed with EORTC QLQ-C30, improved global health status (*p* < .05) and less sleep disturbances (*p* < .05) post intervention as compared to baseline in intervention group		*
Mohammadi, 2022 [59]	EX-PLISSIT, Counselling, four weekly sessions lasting 60–90 min Control group: one online session	Sexual quality of life assessed with the Sexual Quality of Life-Female (SQL-F) questionnaire, indicating higher scores on overall SQL post intervention (ns)		****
Olcer, 2022 [60]	BETTER, Counselling, four sessions with 1-week interval	Quality of life assessed with EORTC-BR23^15^, no significant difference between counselling and control group (ns)	Presence of body image issues assessed with Body Cathexis Scale, no significant difference between counselling and control group (ns)	*****
Perz, 2015 [61]	PLISSIT Self-help booklet consisting of 68 pages providing self-help information (group) or information and 1-h telephone consultation (group 2)	Quality of life assessed with Medical Outcomes Study Health Survey Short Form (SF-12) (ns)	Psychological well-being assessed with Hospital Anxiety and Depression Scale (HADS) (ns)	*
Reese, 2021 [63]	Starting the Conversation, Online patient training including video slideshow (20 min) and five-page workbook	Quality of Life assessed with FACT-B^16^ (ns)	Psychological distress assessed with HADS^17^, indicating reduced anxiety in intervention group as compared to control (*p* < .05)	***
Reese, 2023 [64]	Starting the Conversation, educational intervention for patients including video slideshow (23 min) and five-page workbook		Psychological distress assessed with the Hospital Anxiety and Depressions Scale Anxiety (HADS-A), indicated small improvement regarding anxiety post intervention (no information about significance provided) Depressive symptoms assessed with the Hospital Anxiety and Depression Scale depressive symptoms (HADS-D), indicated no effect post intervention (no information about significance provided)	***
Shalamzari, 2022 [66]	PLISSIT and BETTER-model, Counselling, four sessions of 60–90 min with 1-week interval	Sexual quality of life assessed with Sexual Quality of Life Questionnaire Female (SQoL-F) indicated an increase in quality of life in both groups post intervention as compared to baseline (*p* < .01), with higher scores in the BETTER group. No difference was found between groups in mean changes post intervention as compared to baseline		****
Outcomes related to quality of care				
First author, year	Tool (model, programme, intervention, components, duration)	Satisfaction with communication	Other outcomes related to Quality of Care	MMAT
Bingham, 2022 [40]	Engagement, Assessment, Support and Signposting (EASSi) framework, one-time training (eLearning), duration approximately 1 h		Sexual attitudes and beliefs assessed with the modified Sexual Attitudes and Beliefs Survey (SABS), significant increase post intervention compared to baseline (*p* < .001)	**
McCaughan, 2020 [19]	EASSi^18^ framework (Tablet-based) Training (e-learning) of 30 min to use tool		Sexual well-being attitudes and beliefs, assessed with patient survey, indicating that the tool helped in discussing sexual well-being	**
McCaughan, 2021 [58]	EASSi framework, brief e-learning consisting of three sections		Sexual attitudes and beliefs assessed with the modified Sexual Attitudes and Beliefs Survey (SABS), no significant difference in overall scores after completing the e-learning as compared to baseline (ns)	*
Perz, 2015 [61]	PLISSIT Self-help booklet consisting of 68 pages providing self-help information (group) or information and 1-h telephone consultation (group 2)	Sexual communication assessed with Dyadic Sexual Communication scale (ns)		*
Reese, 2012 [62]	Intimacy Enhancement Intervention, Counselling, four phone-based sessions of 50 min	Sexual communication assessed with Dyadic Sexual Communication Scale, indicating improved communication post intervention as compared to baseline (effect size 0.82)		**
Reese, 2019 [34]	PLISSIT Training to equip clinicians for counselling, two modules 15 and 60 min)	Clinical communication assessed with audio recordings of encounters, indicating an increase in discussing sexual health and an improvement of communication behaviours post intervention as compared to baseline (no information about significance)	Patient satisfaction assessed with the Consumer Satisfaction Index (ns)	*
Reese, 2021 [63]	Starting the Conversation, Online patient training including video slideshow (20 min) and five-page workbook	Clinical communication behaviours assessed with audio recorded clinical encounters, indicating greater odds of asking a question about sexual health in the intervention group as compared to control (OR^19^ 2.85 [1.27–6.38], *p* < .05)		***
Reese, 2023 [64]	Starting the Conversation, educational intervention for patients including video slideshow (23 min) and five-page workbook	Self-efficacy for sexual health communication assessed with two items indicated increased self-efficacy for communication about sexual health post intervention (no information about significance provided)		***
Roberts, 2020 [65]	Brief Sexual Symptom checklist for Women applied in routine care		Referral to sexual counsellor or pelvic floor physiotherapist ascertained from medical records, indicating no significant difference in referrals in intervention group as compared to control	**
Wang, 2022 [68]	EX-PLISSIT applied in training for HCPs^20^	Baseline knowledge and comfort with conversations assessed with HCP survey, indicating an increase in conversations after training (no information about significance provided) Frequency of and satisfaction with conversations about relationship, body image and intimacy assessed through patient survey, indicating conversations in 74% of patients		-
Zhang, 2022 [69]	Structured sessions, Nurse-led intervention, monthly sessions of 4 h, lasting for three consecutive treatment cycles	Marital quality assessed with Olson Marital Quality Questionnaire (ENRICH), indicating post intervention an improved communication in the intervention group as compared to control (*p* < .05) and no significant difference in marital satisfaction or sexual life between intervention and control group (ns)		*****

Abbreviations:

^1^*MMAT* Mixed Methods Appraisal Tool

^2^*PLISSIT* Permission, Limited Information, Specific Suggestions, Intensive Therapy

^3^*P P*-value

^4^*FSFI* Female Sexual Function Index

^5^*NS* not significant

^6^*IIEF* International Index of Erectile Function

^7^*FSFD-R* Female Sexual Distress Scale-Revised

^8^*GSE* Grouped Sexual Education

^9^*EX-PLISSIT* Extended-PLISSIT

^10^*BETTER* Bring up the topic, Explain, Telling, timing, Education, Recording

^11^*PROMIS SexFS* Patient Reported Outcomes Measurement Information System Sexual Functioning and Satisfaction

^12^*WOHQOL-BREF* World Health Organization Quality of Life – Brief Version

^13^*TCHI-FACT-G* Traditional Chinese Version of Functional Assessment of Cancer Therapy-General

^14^*EORTC-QLQ-C30* European Organization for Research and Treatment for Cancer Quality of Life Questionnaire

^15^*EORTC-BR23* European Organization for Research and Treatment of Cancer Quality of life Questionnaire Breast

^16^*FACT-B* Functional Assessment of Cancer Therapy-Breast

^17^*HADS* Hospital Anxiety and Depression Scale

^18^*EASSi* Engagement, Assessment, Support and Signposting

^19^*OR* odds ratio

^20^*HCPs* Healthcare professionals

Sexual function was assessed with the Female Sexual Function Index (FSFI, 13 studies), the Changes in Sexual Functioning Questionnaire (CSFQ-14, one study), the International Index of Erectile Function (IIEF, two studies), the Lasry Sexual Functioning Scale (LSFS, one study), the Patient-Reported Outcomes Measurement Information System Sexual Function and Satisfaction (PROMIS SexFS, one study), the Sexual Health Inventory for Men (SHIM, one study), or a self-developed questionnaire consisting of several items related to sexual function (one study). A statistically significant improvement was found over time in the patient’s sexual functioning in 11 studies, mostly in studies among individual patients. Of these, five studies based their intervention on PLISSIT [38, 48, 50, 54, 59]. One study among couples indicated improvement in female sexual functioning and little improvement in male sexual functioning, without providing information about significance [62]. Six studies did not find a significant difference over time [39, 43, 47, 56, 57, 61].

Sexual distress among individual patients was assessed in two studies, using the Index of Sexual Satisfaction and the Female Sexual Distress Scale-Revised (FSDS-R). Both studies reported a decrease in sexual distress [54, 62]. Of these studies, one study applied PLISSIT-based counselling among individuals [54] and the other study applied a phone-based intervention among couples [62].

Relationship satisfaction was assessed in four studies with the (Brief) Dyadic Adjustment Scale [43, 48, 60, 62] and in one study with the Chinese version of the Revised Dyadic Adjustment Scale (CR-DAY) [56]. Relationship satisfaction significantly improved in one study that used PLISSIT counselling among individuals [48], in one study that applied individual counselling based on the BETTER model [60], and in one study that used a psychosocial intervention among couples [56]. One study, which used a phone-based couple intervention discussing physical intimacy and sexual concerns, indicated a medium-sized effect on relationship satisfaction [62]. This was the only study that reported on intimacy and it indicated little improvement.

#### Outcomes related to quality of life

Nineteen studies reported on outcomes regarding quality of life. Besides overall quality of life (17 studies), studies also reported effects on other related outcomes (six studies), such as sexual quality of life, psychological well-being and body image (see Table 2).

Overall quality of life was assessed using various questionnaires. The assessment was made using the Sexual Quality of Life-Female (SCOL-F, five studies), followed by the European Organization for Research and Treatment for Cancer Quality of Life Questionnaire (EORTC-QLQ-C30, four studies), the World Health Organization Quality of Life Questionnaire-BREF (WHOQoL-BREF, two studies) and the Functional Assessment of Cancer Therapy-General (FACT-G, three studies), the European Organization for Research and Treatment of Cancer Quality of Life Questionnaire Breast (EORTC-QLQ-BR23, one study), the Medical Outcomes Study Health Survey Short Form (SF-12, one study), or a self-developed questionnaire (one study).

In total, seven studies reported a statistically significant improvement over time in overall quality of life [38, 42, 51, 54, 56, 57, 66]. Of these, three studies based their intervention on PLISSIT, which was applied to individual patients [38, 54, 66]. The remaining ten studies did not report a significant difference in overall quality of life. Among these studies, five based their intervention on PLISSIT among individuals (one study) [49], couples (two studies) [50, 61] and groups (three studies) [39, 46, 59].

Psychological well-being was assessed in four studies using the Hospital Anxiety and Depression Scale (HADS, three studies) and the Self-Rating Depression Scale (SDS, one study). A significant reduction in anxiety was found in one study using an educational intervention, based on the principles of social cognitive theory, among individual patients [63]. Another study among individuals with the same intervention reported a reduction in anxiety and depression symptoms, but did not report on significance [64]. Furthermore, in a group of patients receiving an education-based nursing intervention, a decrease in severity of depression was found as compared to the control group [46]. The remaining study, which applied couple-based PLISSIT counselling, did not find a significant difference in psychological well-being [61].

Also, one study that applied PLISSIT-based counselling among individuals [48] reported a significant reduction in treatment-related side effects and a significant improvement in body image, assessed with the Body Image Scale.

#### Outcomes related to quality of care

Eleven studies reported on outcomes regarding quality of care, i.e. satisfaction with communication or other outcomes related to quality of care (see Table 3).

Outcomes related to satisfaction with communication were reported in seven studies. Satisfaction with communication was assessed with the Dyadic Sexual Communication scale (two studies), self-developed items (two studies), audio recordings (two studies) and the Olson Martial Quality Questionnaire (one study). A statistically significant improvement in communication after the intervention was found in only one study, which used a self-developed nurse-led programme among couples [69]. Four studies indicated an improvement in communication, but did not report any information regarding significance [34, 62, 64, 68]. Of these four studies, one study applied PLISSIT in training for healthcare professionals and indicated an increase in discussions of sexual health and improvement of communication behaviours after the intervention [34]. Furthermore, one study reported that there was no significant difference in sexual communication after applying PLISSIT-based counselling among couples [61].

Sexual attitudes were assessed with the Sexual Attitudes and Beliefs Survey (SABS) (two studies) [40, 58] and a patient survey (one study) [19]. Only one study reported a significant improvement after the intervention, which consisted of an eLearning resource for healthcare professionals; it stated that healthcare professionals experienced fewer barriers to providing sexual support [40].

Also, one study among individuals reported on referrals. It found no significant effects of the screening-tool for sexual health on numbers of referrals to a sexual counsellor or pelvic floor physiotherapist, based on patients’ medical records [65].

### Differences in feasibility and effectiveness of communication tools between early stage cancer, advanced cancer and cancer survivors

Of the 35 publications, eight studies focused on cancer survivors [38, 39, 47, 50, 51, 54, 59, 65]. The interventions included in these studies were PLISSIT-based in four studies [39, 50, 54, 59] and based on other tools in the remaining four studies [38, 47, 51, 65]. Furthermore, five of these eight studies involved individual cancer survivors [38, 47, 51, 54, 65], two studies involved groups of patients [39, 59] and one study focused on couples [50]. Willingness to participate was reported in six studies and varied from 40.0% (individual patients) to 90.2% (group of patients) [38, 39, 47, 51, 54, 59]. Lost to follow-up ranged from 3.0 to 57.0% (both were studies of individual patients) [38, 54].

Of these eight studies focussing on cancer survivors, seven studies assessed sexual functioning, predominantly with the Female Sexual Function Index (FSFI). Sexual functioning significantly improved in five studies [38, 50, 51, 54, 59]. Quality of life was assessed in seven studies with different questionnaires; improvement was reported in three studies [38, 51, 54].

None of the studies compared cancer survivors with patients with cancer (early stage or advanced cancer).

## Discussion

This review considered 35 studies on the feasibility and effectiveness of tools to support communication about changes in intimacy and sexuality among patients with cancer or cancer survivors. In 11 studies, the stepwise PLISSIT model was used, while a total of 15 other tools were described in the other studies. The tools were considered feasible and practical and most appeared to be effective in improving sexual functioning, quality of life and quality of care.

Based on the 35 studies, it is not possible to identify any variation in terms of feasibility and effectiveness between patients with early stage cancer and those with advanced cancer. However, previous research has shown that patients with early stage cancer can have different needs compared to patients with advanced cancer [76, 77].

The overall methodological quality of the 35 studies, as assessed with the MMAT tool, varied from low (none of the criteria were met) to high (all criteria were met). The five studies where the methodological quality was assessed as high (four or five criteria were met) used counselling based on PLISSIT, BETTER or Schover’s sexual assessment method and found statistically significant differences in sexual functioning when applied in individual patients [51, 60, 66] or groups of patients [59].

Although our review revealed a broad range of feasible and effective tools, previous research highlighted the reluctance that healthcare professionals might experience when addressing issues related to sexuality and intimacy, resulting in unaddressed issues during care for patients with cancer [17, 25, 26, 28]. Furthermore, earlier research demonstrated that communication tools can be beneficial for healthcare professionals in overcoming barriers and initiating conversations on these sensitive subjects [32–34]. However, due to the wide range of communication tools available and the flexibility in their application, nursing staff or other professionals might find it hard to choose a specific tool. The choice could be based on how often a tool has already been researched and found to be proven effective. This systematic review demonstrates that the PLISSIT-model stands out as both the most commonly researched tool and as an effective method for addressing intimacy and sexuality. Therefore, based on this review, the PLISSIT-model can be recommended.

Additionally, this review shows that of the 35 included studies, only one study addressed effects on intimacy; it indicated little improvement. Most of the research focuses on sexuality among patients with cancer [15, 16, 78]. Studies that focus on outcomes related to sexuality might have adopted an inclusive view that considers sexuality as a fundamental aspect of human being, occasionally encompassing intimacy within its scope [5]. Nonetheless, it is important to recognize that intimacy can be defined as an interactive process in its own right [10, 11].

Another noteworthy finding is that 12 of the studies included in our review were conducted in non-Western countries such as Iran, Turkey, Yemen and Egypt. Various non-Western cultural and religious perspectives might hinder open conversations regarding sexuality [79, 80]. However, none of these studies explicitly address cultural or religious factors. Nevertheless, it is recommended for healthcare professionals to be aware of cultural and religious aspects in their communication, especially in communication about the end of life [81].

## Methodological considerations

A strength of this systematic review is that we used multiple relevant literature databases in the search process. An additional strength is that we performed a methodological appraisal of the studies that were included. The MMAT tool enabled us to assess both quantitative and qualitative research designs.

A limitation of this review is the fact that the included studies considered various outcomes and used a variety of questionnaires to assess the outcomes. Most used questionnaires are previously validated, such as FSFI, EORT-QLQ and HADS. However, because of the various outcomes and variety of questionnaires, we were unable to conduct a meta-analysis. Nevertheless, it was evident that the overall use of communication tools proved to be effective.

Another limitation of this review is that we had to exclude five articles that were written in Korean or Chinese and that seemed to fulfil our inclusion criteria [70–74]. Four of these studies applied PLISSIT in their intervention [71–74] and one study used a Sexual Health Improvement Programme [70]. Results regarding sexual functioning could however be read from tables and abstracts; all reported significant improvement in sexual functioning, which seems to be in line with the findings of our review [70–74].

## Conclusion

This systematic review shows that the use of tools that support communication about changes in intimacy and sexuality among individual patients with cancer or cancer survivors, couples or groups of patients is feasible and effective. Various communication tools were used in counselling, educational or nurse-led programmes. The most commonly used tool, the stepwise PLISSIT model, proved to be feasible for HCPs and to have a positive effect on patients’ and partners’ sexual functioning and quality of life. However, the specific communication tool or approach utilized seems to matter less than the very fact of paying attention to changes in intimacy and sexuality.

### Supplementary Information

Below is the link to the electronic supplementary material.

Supplementary file1 (DOCX 21 KB)

Supplementary file2 (DOCX 17 KB)

Supplementary file3 (DOCX 19 KB)
