# Supplementary material for: Feasibility and effectiveness of communication tools for addressing intimacy and sexuality in patients with cancer: a systematic review
Source: Support Care Cancer. 2024 Jan 17;32(2):109. doi: 10.1007/s00520-024-08308-6 (PMC10794301; doi:10.1007/s00520-024-08308-6)
Supplement: Supplementary file 1 — Supplementary file1 (DOCX 21 KB) [file 520_2024_8308_MOESM1_ESM.docx]

**Feasibility and effectiveness of communication tools for addressing intimacy and sexuality in patients with cancer: a systematic review**

Susanne A. M. Arends, Carlijn E. van Rossum, Corien M. Eeltink, Jantien E. Robertus, Linda J. Schoonmade, Anneke L. Francke, Irene P. Jongerden

*Journal submission: Supportive Care in Cancer*

**Corresponding author:**Susanne Arends, Amsterdam UMC, Vrije Universiteit Amsterdam, Department of Public and Occupational Health. Van der Boechorststraat 7, NL-1081 BT Amsterdam, The Netherlands.
E-mail: [s.a.arends@amsterdamumc.nl](mailto:s.a.arends@amsterdamumc.nl)

See supplemental material on the next page.

Supplemental materials I

**Table presenting the results of the quality assessment^1^**

| **First author, year** | **Criteria from the Mixed Methods Appraisal Tool^3^** | | | | | | | | | | | | | | | | | | | | | | | | |
| --- | --- | --- | --- | --- | --- | --- | --- | --- | --- | --- | --- | --- | --- | --- | --- | --- | --- | --- | --- | --- | --- | --- | --- | --- | --- |
|  | **1. Qualitative studies** | | | | | **2. Randomized controlled trials** | | | | | **3. Non-randomized studies** | | | | | **4. Quantitative descriptive studies** | | | | | **5. Mixed methods studies** | | | | |
|  | 1.1 | 1.2 | 1.3 | 1.4 | 1.5 | 2.1 | 2.2 | 2.3 | 2.4 | 2.5 | 3.1 | 3.2 | 3.3 | 3.4 | 3.5 | 4.1 | 4.2 | 4.3 | 4.4 | 4.5 | 5.1 | 5.2 | 5.3 | 5.4 | 5.5 |
| Akeflo, 2022 |  |  |  |  |  |  |  |  |  |  |  |  |  |  |  |  |  |  |  |  |  |  |  |  |  |
| De Almeida, 2020 |  |  |  |  |  |  |  |  |  |  |  |  |  |  |  |  |  |  |  |  |  |  |  |  |  |
| Bingham, 2022 |  |  |  |  |  |  |  |  |  |  |  |  |  |  |  |  |  |  |  |  |  |  |  |  |  |
| Bokaie, 2022 |  |  |  |  |  |  |  |  |  |  |  |  |  |  |  |  |  |  |  |  |  |  |  |  |  |
| Bokaie, 2023 |  |  |  |  |  |  |  |  |  |  |  |  |  |  |  |  |  |  |  |  |  |  |  |  |  |
| Chambers, 2015 |  |  |  |  |  |  |  |  |  |  |  |  |  |  |  |  |  |  |  |  |  |  |  |  |  |
| Chow,  2014^2^ |  |  |  |  |  |  |  |  |  |  |  |  |  |  |  |  |  |  |  |  |  |  |  |  |  |
| Cullen, 2021 |  |  |  |  |  |  |  |  |  |  |  |  |  |  |  |  |  |  |  |  |  |  |  |  |  |
| Du., 2020 |  |  |  |  |  |  |  |  |  |  |  |  |  |  |  |  |  |  |  |  |  |  |  |  |  |
| DuHamel, 2016 |  |  |  |  |  |  |  |  |  |  |  |  |  |  |  |  |  |  |  |  |  |  |  |  |  |
| El-Sayed Saboula, 2015 |  |  |  |  |  |  |  |  |  |  |  |  |  |  |  |  |  |  |  |  |  |  |  |  |  |
| Esmkhani, 2021 |  |  |  |  |  |  |  |  |  |  |  |  |  |  |  |  |  |  |  |  |  |  |  |  |  |
| Faghani, 2016 |  |  |  |  |  |  |  |  |  |  |  |  |  |  |  |  |  |  |  |  |  |  |  |  |  |
| Fatehi., 2019 |  |  |  |  |  |  |  |  |  |  |  |  |  |  |  |  |  |  |  |  |  |  |  |  |  |
|  | 1.1 | 1.2 | 1.3 | 1.4 | 1.5 | 2.1 | 2.2 | 2.3 | 2.4 | 2.5 | 3.1 | 3.2 | 3.3 | 3.4 | 3.5 | 4.1 | 4.2 | 4.3 | 4.4 | 4.5 | 5.1 | 5.2 | 5.3 | 5.4 | 5.5 |
| Jonsdottir, 2021 |  |  |  |  |  |  |  |  |  |  |  |  |  |  |  |  |  |  |  |  |  |  |  |  |  |
| Jonsdottir, 2021 |  |  |  |  |  |  |  |  |  |  |  |  |  |  |  |  |  |  |  |  |  |  |  |  |  |
| Keshavarz, 2021 |  |  |  |  |  |  |  |  |  |  |  |  |  |  |  |  |  |  |  |  |  |  |  |  |  |
| Khoei., 2022 |  |  |  |  |  |  |  |  |  |  |  |  |  |  |  |  |  |  |  |  |  |  |  |  |  |
| Li, 2023 |  |  |  |  |  |  |  |  |  |  |  |  |  |  |  |  |  |  |  |  |  |  |  |  |  |
| Maughan, 2001^2^ |  |  |  |  |  |  |  |  |  |  |  |  |  |  |  |  |  |  |  |  |  |  |  |  |  |
| McCaughan, 2020^2^ |  |  |  |  |  |  |  |  |  |  |  |  |  |  |  |  |  |  |  |  |  |  |  |  |  |
| McCaughan, 2021^2^ |  |  |  |  |  |  |  |  |  |  |  |  |  |  |  |  |  |  |  |  |  |  |  |  |  |
| Mohammadi, 2022 |  |  |  |  |  |  |  |  |  |  |  |  |  |  |  |  |  |  |  |  |  |  |  |  |  |
| Olcer, 2022 |  |  |  |  |  |  |  |  |  |  |  |  |  |  |  |  |  |  |  |  |  |  |  |  |  |
| Perz, 2015 |  |  |  |  |  |  |  |  |  |  |  |  |  |  |  |  |  |  |  |  |  |  |  |  |  |
| Reese, 2012 |  |  |  |  |  |  |  |  |  |  |  |  |  |  |  |  |  |  |  |  |  |  |  |  |  |
| Reese, 2019^2^ |  |  |  |  |  |  |  |  |  |  |  |  |  |  |  |  |  |  |  |  |  |  |  |  |  |
| Reese, 2021 |  |  |  |  |  |  |  |  |  |  |  |  |  |  |  |  |  |  |  |  |  |  |  |  |  |
| Reese, 2023 |  |  |  |  |  |  |  |  |  |  |  |  |  |  |  |  |  |  |  |  |  |  |  |  |  |
| Roberts, 2020 |  |  |  |  |  |  |  |  |  |  |  |  |  |  |  |  |  |  |  |  |  |  |  |  |  |
| Shalamzari, 2022 |  |  |  |  |  |  |  |  |  |  |  |  |  |  |  |  |  |  |  |  |  |  |  |  |  |

|  | 1.1 | 1.2 | 1.3 | 1.4 | 1.5 | 2.1 | 2.2 | 2.3 | 2.4 | 2.5 | 3.1 | 3.2 | | 3.3 | 3.4 | 3.5 | 4.1 | 4.2 | 4.3 | 4.4 | 4.5 | 5.1 | 5.2 | 5.3 | 5.4 | 5.5 |
| --- | --- | --- | --- | --- | --- | --- | --- | --- | --- | --- | --- | --- | --- | --- | --- | --- | --- | --- | --- | --- | --- | --- | --- | --- | --- | --- |
| Taleb, 2023 |  |  |  |  |  |  |  |  |  |  |  |  | |  |  |  |  |  |  |  |  |  |  |  |  |  |
| Wang, 2022^2^ |  |  |  |  |  |  |  |  |  |  |  |  | |  |  |  |  |  |  |  |  |  |  |  |  |  |
| Winterling, 2020 |  |  |  |  |  |  |  |  |  |  |  |  | |  |  |  |  |  |  |  |  |  |  |  |  |  |
| Zhang, 2022 |  |  |  |  |  |  |  |  |  |  |  |  | |  |  |  |  |  |  |  |  |  |  |  |  |  |
| *^1^Studies were appraised following the criteria from the mixed methods appraisal tool (MMAT) and presented per study. Green colour = study meets the criteria, red colour = study does not meet the criteria, orange colour = can’t tell if the study meets the criteria. We determined the overall scores of each study based on the number of green boxes.* | | | | | | | | | | | | | | | | | | | | | | | | | | |
| *^2^ Mixed methods studies were appraised following the algorithm for selecting the study categories to rate in the MMAT. The design with the lowest score was accounted in the study. Adapted from: National Institute for Health Care Excellence. (2012). Methods for the development of nice public health guidance. London: National Institute for Health and Care Excellence; and Scottish Intercollegiate Guidelines Network. (2017). Algorithm for classifying study design for questions of effectiveness. Retrieved December 1, 2017, from http://www.sign.ac.uk/assets/study_design.pdf.* | | | | | | | | | | | | | | | | | | | | | | | | | | |
| *^3^ Methodological quality criteria according to MMAT tool:* 1.1. Is the qualitative approach appropriate to answer the research question?  1.2. Are the qualitative data collection methods adequate to address the research question?  1.3. Are the findings adequately derived from the data?  1.4. Is the interpretation of results sufficiently substantiated by data?  1.5. Is there coherence between qualitative data sources, collection, analysis and interpretation?  2.1. Is randomization appropriately performed?  2.2. Are the groups comparable at baseline?  2.3. Are there complete outcome data?  2.4. Are outcome assessors blinded to the intervention provided?  2.5. Did the participants adhere to the assigned intervention?  3.1. Are the participants representative of the target population?  3.2. Are measurements appropriate regarding both the outcome and intervention (or exposure)?  3.3. Are there complete outcome data? | | | | | | | | | | | | | 3.4. Are the confounders accounted for in the design and analysis?  3.5. During the study period, is the intervention administered (or exposure occurred) as intended?  4.1. Is the sampling strategy relevant to address the research question?  4.2. Is the sample representative of the target population?  4.3. Are the measurements appropriate?  4.4. Is the risk of nonresponse bias low?  4.5. Is the statistical analysis appropriate to answer the research question?  5.1. Is there an adequate rationale for using a mixed methods design to address the research question?  5.2. Are the different components of the study effectively integrated to answer the research question?  5.3. Are the outputs of the integration of qualitative and quantitative components adequately interpret?  5.4. Are divergences and inconsistencies between quantitative and qualitative results adequately addressed?  5.5. Do the different components of the study adhere to the quality criteria of each tradition of the methods involved? | | | | | | | | | | | | | |
