# Supplementary material for: Feasibility and effectiveness of communication tools for addressing intimacy and sexuality in patients with cancer: a systematic review
Source: Support Care Cancer. 2024 Jan 17;32(2):109. doi: 10.1007/s00520-024-08308-6 (PMC10794301; doi:10.1007/s00520-024-08308-6)
Supplement: Supplementary file 2 — Supplementary file2 (DOCX 17 KB) [file 520_2024_8308_MOESM2_ESM.docx]

**Feasibility and effectiveness of communication tools for addressing intimacy and sexuality in patients with cancer: a systematic review**

Susanne A. M. Arends, Carlijn E. van Rossum, Corien M. Eeltink, Jantien E. Robertus, Linda J. Schoonmade, Anneke L. Francke, Irene P. Jongerden

*Journal submission: Supportive Care in Cancer*

**Corresponding author:**Susanne Arends, Amsterdam UMC, Vrije Universiteit Amsterdam, Department of Public and Occupational Health. Van der Boechorststraat 7, NL-1081 BT Amsterdam, The Netherlands.
E-mail: [s.a.arends@amsterdamumc.nl](mailto:s.a.arends@amsterdamumc.nl)

See supplemental material on the next page.

Supplemental materials II

**Table overview tools included studies**

| **Tools - individual patients** | | |
| --- | --- | --- |
| Åkeflo, 2022 | PLISSIT^1^ | Nurse-led intervention |
| El-Sayed Saboula, 2015 | PLISSIT | Counselling sessions (6 sessions, 2 hrs) |
| Esmkhani, 2021^*^ | PLISSIT | Counselling sessions (1-3 sessions) |
| Keshavarz, 2021 | PLISSIT | Counselling sessions (7 sessions, 60 min) |
| Khoei, 2020^*^ | PLISSIT | Counselling sessions (1-3 sessions, 40-60 min) |
| Shalamzari, 2022^*^ | PLISSIT  BETTER model | Counselling sessions (4 sessions, 60-90 min) |
| Fatehi, 2019 | Schover’s sexual assessment method | Psycho-sexual counselling sessions (6 weekly sessions, 90-120 min) |
| Maughan, 2001 | Clinical Nurse Specialist Intervention, including information, advise and support | Counselling sessions (no limit, average 3 home visits) |
| Olcer, 2022 | BETTER^2^ model, including booklet & CD | Counselling sessions (4 sessions, 45-60 min) |
| Chow, 2014 | Self-developed, based on previous study | Educational program (3 individual sessions, 1 group session) |
| DuHamel, 2016 | Self-developed, based on prior research and experiences from investigators | Educational intervention (CSI-SH^3^) (4 sessions, 1 hrs & booster calls) |
| Reese, 2023 | Based on principles of social cognitive theory, including video slideshow + workbook + resource guide | Educational intervention (STC^4^) |
| Reese, 2021 | Based on principles of social cognitive theory, including video slideshow + workbook + skills training | Educational intervention (STC) |
| Taleb., 2023 | Information and advise | Nursing intervention |
| Roberts, 2020 | Brief Sexual Symptom checklist for women | Routine care |
| **Tools - couples** | | |
| Faghani, 2016 | PLISSIT | Counselling sessions (4 sessions, 90 min) |
| Perz, 2015 | PLISSIT (including booklet and consultation) | Counselling sessions |
| Cullen, 2021 | Self-developed, based on Acceptance and Commitment Therapy and the Physical Pleasure-Relational Intimacy Model of Sexual Motivation | Online psycho-sexual intervention (iRISE^5^) |
| Chambers, 2015 | Based on cognitive behavioral sex and couples therapy | Counselling by nurses or peers (2-8 sessions) |
| Reese, 2012 | Phone based using techniques from sex therapy and couple/marital therapy | Intimacy enhancement intervention program (4 sessions, 50 min) |
| Jonsdottir, 2021 | CO-SOTC^6^ intervention based on Illness Beliefs Model (IBM) | Counselling sessions (3 sessions, 45 min) |
| Jonsdottir, 2021 | CO-SOTC intervention based on Illness Beliefs Model (IBM) | Counselling sessions (3 sessions, 45 min) |
| Li, 2023 | Systematic Transactional Model of Stress and Coping | Psychosocial intervention program (WeChat) (8 week program) |
| Zhang, 2022 | Self-developed based on iterative discussions and consultations with a multidisciplinary expert team. | Nurse-led program (monthly sessions of 4 hrs) |
| **Tools – groups of patients** | | |
| Almeida, 2020 | PLISSIT | Counselling Sessions (5 weekly sessions, 90 min) |
| Mohammadi, 2022 | EX-PLISSIT^7^ | Counselling sessions (4 weekly sessions, 60-90 min) |
| Bokaie, 2022 | Problem-solving approach | Counselling sessions (8 sessions, 90 min) |
| Bokaie, 2023 | Solution-focused approach | Counselling sessions (8 weekly online sessions, 90 min) |
| Esmkhani, 2021^*^ | Sexual Health Model (SHM) | Educational intervention (6 hr workshop and 10 sessions) |
| Du, 2020 | Nursing interventions & empowerment education based nursing interventions | Educational sessions (7 sessions of at least 30 minutes) |
| Khoei, 2022^*^ | Grouped Sexuality Education (GSE) | Educational intervention (6 hr workshop and 10 sessions) |
| **Tools – HCP (predominantly nurses) to improve communication with individual patients** | | |
| Wang, 2022 | EX-PLISSIT | RBI pilot and MRT training |
| Reese, 2019 | Self-study and workshop including informational workbook and supplementary materials based on social cognitive theory and previous research. | Education to equip HCP for PLISSIT counselling (iShare^8^) |
| Bingham, 2022 | EASSi^9^ framework | eLearning resource (1-time training, approx. 1 hr) |
| McCaughan, 2020 | EASSi framework | Tablet-based training tool (30 min training program) |
| McCaughan, 2021 | EASSi framework | Brief e-learning (3 sections) |
| Winterling, 2020 | Video, lecture, role-play and homework assignment | Educational intervention (Fex-talk) (single session, 2 hrs) |
| ^*^Multiple tools assessed in one study | |  |
| Abbreviations: ^1^PLISSIT: Permission, Limited Information, Specific Suggestions, Intensive Therapy  ^2^BETTER: Bring up the topic, Explain, Telling, Timing, Education, Recording  ^3^CSI-SH: Cancer survivorship Intervention-Sexual Health  ^4^STC: Starting the Conversation  ^5^iRISE: Online relational intimacy and sexual enhancement intervention  ^6^CO-SOTC: Couple Strengths-Oriented Therapeutic Conversation  ^7^EX-PLISSIT: Extended PLISSIT (Permission, Limited Information, Specific Suggestions, Intensive Therapy)  ^8^iSHARE: Improving Sexual Health and Augmenting Relationships through Education  ^9^EASSI: Engagement, Assessment, Support and Signposting | | |
