# Supplementary material for: Feasibility and effectiveness of communication tools for addressing intimacy and sexuality in patients with cancer: a systematic review
Source: Support Care Cancer. 2024 Jan 17;32(2):109. doi: 10.1007/s00520-024-08308-6 (PMC10794301; doi:10.1007/s00520-024-08308-6)
Supplement: Supplementary file 3 — Supplementary file3 (DOCX 19 KB) [file 520_2024_8308_MOESM3_ESM.docx]

**Feasibility and effectiveness of communication tools for addressing intimacy and sexuality in patients with cancer: a systematic review**

Susanne A. M. Arends, Carlijn E. van Rossum, Corien M. Eeltink, Jantien E. Robertus, Linda J. Schoonmade, Anneke L. Francke, Irene P. Jongerden

*Journal submission: Supportive Care in Cancer*

**Corresponding author:**Susanne Arends, Amsterdam UMC, Vrije Universiteit Amsterdam, Department of Public and Occupational Health. Van der Boechorststraat 7, NL-1081 BT Amsterdam, The Netherlands.
E-mail: [s.a.arends@amsterdamumc.nl](mailto:s.a.arends@amsterdamumc.nl)

See supplemental material on the next page.

Supplemental materials III

# search june 22, 2023

### 3.1.1. PubMed Search History June 22, 2023

| **Search** | **PubMed Query – June 22, 2023** | **Results** |
| --- | --- | --- |
| #5 | #4 NOT (("Adolescent"[Mesh] OR "Child"[Mesh] OR "Infant"[Mesh] OR adolescen*[tiab] OR child*[tiab] OR schoolchild*[tiab] OR infant*[tiab] OR girl*[tiab] OR boy[tiab] OR boys[tiab] OR boyhood[tiab] OR teen[tiab] OR teens[tiab] OR teenager*[tiab] OR youth*[tiab] OR pediatr*[tiab] OR paediatr*[tiab] OR puber*[tiab]) NOT ("Adult"[Mesh] OR adult*[tiab] OR man[tiab] OR men[tiab] OR woman[tiab] OR women[tiab])) | 2,456 |
| #4 | #1 AND #2 AND #3 | 2,577 |
| #3 | "Communication"[Mesh] OR "Counseling"[Mesh] OR communicati*[tiab] OR counseling[tiab] OR counselling[tiab] OR consult*[tiab] OR conversation*[tiab] OR ((PLISSIT[tiab] OR ALARM[tiab] OR PLEASURE[tiab]) AND (model*[tiab] OR framework*[tiab] OR tool*[tiab])) OR "BETTER model"[tiab] | 940,736 |
| #2 | "Sexuality"[Mesh:NoExp] OR "Sexual Health"[Mesh] OR sexual*[tiab] OR intimacy[tiab] OR intimate[tiab] | 313,240 |
| #1 | "Neoplasms"[Mesh] OR cancer*[tiab] OR tumor*[tiab] OR tumour*[tiab] OR neoplasm*[tiab] OR malign*[tiab] OR oncolog*[tiab] | 5,018,226 |

### 3.1.2. Search History Embase June 22, 2023

| **Search** | **Embase.com Query – June 22, 2023** | **Results** |
| --- | --- | --- |
| #6 | #5 NOT ('conference abstract'/it OR 'conference review'/it) | 3,702 |
| #5 | #4 NOT (('adolescent'/exp OR 'child'/exp OR adolescent*:ti,ab,kw OR child*:ti,ab,kw OR schoolchild*:ti,ab,kw OR infant*:ti,ab,kw OR girl*:ti,ab,kw OR boy*:ti,ab,kw OR teen:ti,ab,kw OR teens:ti,ab,kw OR teenager*:ti,ab,kw OR youth*:ti,ab,kw OR pediatr*:ti,ab,kw OR paediatr*:ti,ab,kw OR puber*:ti,ab,kw ) NOT ('adult'/exp OR 'aged'/exp OR 'middle aged'/exp OR adult*:ti,ab,kw OR man:ti,ab,kw OR men:ti,ab,kw OR woman:ti,ab,kw OR women:ti,ab,kw)) | 5,926 |
| #4 | #1 AND #2 AND #3 | 6,261 |
| #3 | 'interpersonal communication'/exp OR 'counseling'/exp OR (communicati* OR counseling OR counselling OR consult* OR conversation* OR ((PLISSIT OR ALARM OR PLEASURE) AND (model* OR framework* OR tool*)) OR ‘BETTER model’):ti,ab,kw | 1,616,995 |
| #2 | 'sexuality'/de OR 'sexual health'/exp OR (sexual* OR intimacy OR intimate):ti,ab,kw | 406,541 |
| #1 | 'neoplasm'/exp OR (cancer* OR tumor* OR tumour* OR neoplasm* OR malign* OR oncolog*):ti,ab,kw | 7,0980,093 |

### 3.1.3. Search History cinahl (ebsco) June 22, 2023

| **Search** | **Cinahl (Ebsco) Query – June 22, 2023** | **Results** |
| --- | --- | --- |
| S5 | S4 NOT ((ZG ("adolescence (13-17 yrs)" OR "childhood (birth-12 yrs)" OR "infancy (2-23 mo)” OR "neonatal (birth-1 mo)" OR "preschool age (2-5 yrs)" OR "school age (6-12 yrs)") OR TI (adolescen* OR child* OR schoolchild* OR infant* OR girl* OR boy* OR teen OR teens OR teenager* OR youth* OR pediatr* OR paediatr* OR puber*) OR AB (adolescen* OR child* OR schoolchild* OR infant* OR girl* OR boy* OR teen OR teens OR teenager* OR youth* OR pediatr* OR paediatr* OR puber*)) NOT (ZG ("adulthood (18 yrs & older)" OR "aged (65 yrs & older)" OR "middle age (40-64 yrs)" OR "thirties (30-39 yrs)" OR "very old (85 yrs & older)") OR TI (adult* OR man OR men OR woman OR women) OR AB (adult* OR man OR men OR woman OR women))) | 1,328 |
| S4 | S1 AND S2 AND S3 | 1,400 |
| S3 | MH ("Communication+" OR "Counseling+") OR TI (communicati* OR counseling OR counselling OR consult* OR conversation* OR ((PLISSIT OR ALARM OR PLEASURE) AND (model* OR framework* OR tool*)) OR “BETTER model”)) OR AB (communicati* OR counseling OR counselling OR consult* OR conversation* OR ((PLISSIT OR ALARM OR PLEASURE) AND (model* OR framework* OR tool*)) OR "BETTER model")) | 551,277 |
| S2 | MH ("Sexuality" OR "Sexual Health") OR TI (sexual* OR intimacy OR intimate) OR AB (sexual* OR intimacy OR intimate) | 124,166 |
| S1 | MH "Neoplasms+" OR TI (cancer* OR tumor* OR tumour* OR neoplasm* OR malign* OR oncolog*) OR AB (cancer* OR tumor* OR tumour* OR neoplasm* OR malign* OR oncolog*) | 888,164 |

### 3.1.4. Search History psycInfo (ebsco) June 22, 2023

| **Search** | **APA PsycInfo Query – June 22, 2023** | **Results** |
| --- | --- | --- |
| S5 | S4 NOT ((ZG ("adolescence (13-17 yrs)" OR "childhood (birth-12 yrs)" OR "infancy (2-23 mo)” OR "neonatal (birth-1 mo)" OR "preschool age (2-5 yrs)" OR "school age (6-12 yrs)") OR TI (adolescen* OR child* OR schoolchild* OR infant* OR girl* OR boy* OR teen OR teens OR teenager* OR youth* OR pediatr* OR paediatr* OR puber*) OR AB (adolescen* OR child* OR schoolchild* OR infant* OR girl* OR boy* OR teen OR teens OR teenager* OR youth* OR pediatr* OR paediatr* OR puber*)) NOT (ZG ("adulthood (18 yrs & older)" OR "aged (65 yrs & older)" OR "middle age (40-64 yrs)" OR "thirties (30-39 yrs)" OR "very old (85 yrs & older)") OR TI (adult* OR man OR men OR woman OR women) OR AB (adult* OR man OR men OR woman OR women))) | 721 |
| S4 | S1 AND S2 AND S3 | 750 |
| S3 | DE ("Communication" OR "Conversation" OR "Cross Cultural Communication" OR "Communication Skills" OR "Counseling") OR TI (communicati* OR counseling OR counselling OR consult* OR conversation* OR ((PLISSIT OR ALARM OR PLEASURE) AND (model* OR framework* OR tool*)) OR ‘BETTER model’)) OR AB (communicati* OR counseling OR counselling OR consult* OR conversation* OR ((PLISSIT OR ALARM OR PLEASURE) AND (model* OR framework* OR tool*)) OR “BETTER model”)) OR KW (communicati* OR counseling OR counselling OR consult* OR conversation* OR ((PLISSIT OR ALARM OR PLEASURE) AND (model* OR framework* OR tool*)) OR “BETTER model”)) | 429,349 |
| S2 | DE ("Sexuality" OR "Sexual Health") OR TI (sexual* OR intimacy OR intimate) OR AB (sexual* OR intimacy OR intimate) OR KW (sexual* OR intimacy OR intimate) | 231,877 |
| S1 | DE ("Neoplasms" OR "Benign Neoplasms" OR "Breast Neoplasms" OR "Endocrine Neoplasms" OR "Leukemias" OR "Melanoma" OR "Metastasis" OR "Nervous System Neoplasms" OR "Terminal Cancer") OR TI (cancer* OR tumor* OR tumour* OR neoplasm* OR malign* OR oncolog*) OR AB (cancer* OR tumor* OR tumour* OR neoplasm* OR malign* OR oncolog*) OR KW (cancer* OR tumor* OR tumour* OR neoplasm* OR malign* OR oncolog*) | 98,148 |

### 3.1.5. Search History Web of Science Core Collection June 22, 2023

| **Search** | **Web of Science Core Collection Query – June 22, 2023** | **Results** |
| --- | --- | --- |
| #4 | #1 AND #2 AND #3 | 2,579 |
| #3 | TS= (communicati* OR counseling OR counselling OR consult* OR conversation* OR ((PLISSIT OR ALARM OR PLEASURE) AND (model* OR framework* OR tool*)) OR “BETTER model”) | 1,279,542 |
| #2 | TS = (sexual* OR intimacy OR intimate) | 491,872 |
| #1 | TS = (cancer* OR tumor* OR tumour* OR neoplasm* OR malign* OR oncolog*) | 4,234,362 |

### 3.1.6. Search History the Cochrane Library June 22, 2023

| **Search** | **The Cochrane Library (Wiley) Query – June 22, 2023** | **Results** |
| --- | --- | --- |
| #4 | #1 and #2 and #3 | 483 |
| #3 | (communicati* OR counseling OR counselling OR consult* OR conversation* OR ((PLISSIT OR ALARM OR PLEASURE) AND (model* OR framework* OR tool*)) OR “BETTER model”):ti,ab,kw  (word variations have been searched) | 75,408 |
| #2 | (sexual* OR intimacy OR intimate):ti,ab,kw  (word variations have been searched) | 26,243 |
| #1 | (cancer* OR tumor* OR tumour* OR neoplasm* OR malign* OR oncolog*):ti,ab,kw  (word variations have been searched) | 257,903 |
